# Supplementary material for: alms1 mutant zebrafish do not show hair cell phenotypes seen in other cilia mutants
Source: PLoS One. 2021 Apr 1;16(4):e0246844. doi: 10.1371/journal.pone.0246844 (PMC8016283; doi:10.1371/journal.pone.0246844)
Supplement: Attachment — Submitted filename: Reviewer Response.docx [file pone.0246844.s004.docx]

Below please find our response to the concerns brought up by the reviewers. We have put our responses in blue.

**Reviewer #1**

Major concerns
• The authors demonstrate that zebrafish alms1 mutants lack phenotypes typically associated with hair cell dysfunction and speculate that genetic compensation, lack of conservation with the mammalian homolog, or for a different role in different hair cell types. What the authors have not done, however, is show that alms1 is expressed in lateral line hair cells during development. If alms1 isn’t expressed in lateral line hair cells, then the absence of a phenotype would not be surprising.

We have investigated existing transcriptome data from zebrafish hair cells to confirm *alms1* expression. We found it was identified as being expressed in studies that looked at the total hair cell transcriptome in both larvae and adults and also clustered with mature hair cells in a lateral line specific single cell RNA dataset. We have added this information to the discussion (lines 313-316).

• More information should also be included that discusses potential paralogs, including a rigorous homology search for any paralog/homologs that may not be listed in Ensembl.

Searching for known ohnologues of *alms1* that may have resulted from the genome duplication in teleost fish did not identify any hits. We also performed searches for homologous proteins and found only one, a C10orf90 homologue which is also a known mammalian ALMS motif containing gene. We have added this information to the discussion (lines 336-345).

• Lines 170-172: While the authors state that cilia look grossly normal, additional quantitative metrics would strengthen their argument. Are cilia lengths normal? Is there some way to assess cilia polarity or the degree of splaying that occurs in other mutants? While the authors are likely correct, having additional evidence that is less subjective would add rigor.

We have added quantification of cilia length for all cell types we examined. This is in the new version of Figure 1.

• To more fully support the argument that alms1 may have a role in auditory hair cells, the authors should examine cilia during auditory hair cell development or maintenance in alms1 larvae by imaging cilia within the otic vesicle (see early work from Jarema Malicki or Bruce Riley for examples).

We imaged the otic vesicle during development as the viewer suggested and included this in the new version of Figure 1.

Minor concerns
• Line 127: please correct the dilution value for the otoferlin antibody.

We have corrected this. The antibody was diluted at 1:100.

**Reviewer #2**

- Even if the final message of this publication is the lack of phenotype, one concern is the absence of additional analysis. As an example, in figure 1, I would appreciate to know the number of ciliated cells even if change will not be observed. Also, authors should draw arrows to point the lateral line and cells of the olfactory pit in both control and Alms1 mutant zebrafish.

We have quantified the length of the cilia and noted the number of animals we did this in. The close contact of the cells we are examining at 5dpf make it difficult to distinguish which cilia come from which cell to get an exact cilia/cell count. However, in the 24 hpf fish where we are only looking at 2 ciliated hair cells in the otic vesicle we do not note any cells without cilia which we have noted in the results section (lines 191-193).

We have also added arrows and brackets to figure 1 to label the cilia of the hair cells in 1A & 1B. We were not sure how to do this for the olfactory pit as the entire structure imaged is the olfactory pit.

- Microscopy material and method details associated with the antibody staining are lacking in the method paragraph. Number of cells/cilia counted should also be precise.

We have changed the “antibody labeling” methods section to “antibody labeling, imaging, and cilia length measurement” and included details about how the images were generated (lines 130-133). As we have quantified cilia length as mentioned above we also have the information as to the number of animals we did that for in the figure legend for that data.

- Escaping approach used to study mechanotransduction is not described in the method and no quantification of the escaping movement is provided with the supplemental video.

We have added a section in the methods on the techniques used for “vibrational stimulus response” (lines 237-241). Additionally, we have quantified the number of fish that show a response and included this as a new Figure 2.

- Discussion paragraph on the lack of conservation with the N terminal mammalian isoform is pretty unclear and might need to be clarified or reformulated.

We have rewritten that section as follows “It is also possible that the lack of phenotype we observe is due to a lack of sequence conservation between the mammalian and zebrafish *alms1* gene. The majority of the N-terminus of the human *ALMS1* gene, encoding for the tandem repeat domain of the protein, is specific to mammals [67,68] with the first 1560 amino acids of the human gene having no homologous region in the zebrafish gene.” (lines 324-331)
